# Process of Attack on Cashew Tree Branches by *Diastocera trifasciata* (Coleoptera: Cerambycidae) and the Relationship between These Attacks and the Phenological Stages in the Gbêkê Region (Central Côte d’Ivoire)

**DOI:** 10.3390/insects11080456

**Published:** 2020-07-22

**Authors:** San-Whouly M. Ouali-N’Goran, Ettien Narcice Akessé, Gniré Mariam Ouattara, Daouda Koné

**Affiliations:** 1 Laboratory of Zoology and Animal Biology, Biosciences Training and Research Unit, Félix Houphouët-Boigny University, 22 BP 582 Abidjan 22, Cote D’Ivoire; narcice1985@gmail.com; 2 African Center of Excellence on Climate Change, Biodiversity and Sustainable Agriculture, Ex ESIE Bingerville-scientific pole of Félix Houphouët-Boigny University, 22 BP 582 Abidjan 22, Cote D’Ivoire; daoudakone2013@gmail.com; 3 Economics and Development Training and Research Unit, Alassane Ouattara University, BP V 18 Bouaké 01, Cote D’Ivoire; gniremariam@conseilcotonanacarde.ci; 4 Cashew and Cotton Council of Côte D’Ivoire, 27 BP 604 Abidjan 27, Cote D’Ivoire; 5 Laboratory of Plant Physiology, Biosciences Training and Research Unit, Félix Houphouët-Boigny University, 22 BP 582 Abidjan 22, Cote D’Ivoire

**Keywords:** attacks, *Diastoceratrifasciata*, Cerambycidae, cashew, branches girdler, phenological stages, Côte d’Ivoire

## Abstract

Cerambycidae *Diastocera trifasciata* attacks were studied from October 2015 to September 2017 in three cashew tree orchards in the locality of Brobo in central Côte d’Ivoire. One hundred fifty-three (153) cashew trees, arranged on a diagonal from each orchard, were selected for sampling. The attacked plants and the branches cut per tree were counted every 15 days. Biotic parameters, namely phenological stages of trees, and abiotic factors, which are rainfall, relative humidity and average temperature, were recorded throughout the study. Attacks were observed from mid-September to January from the pre-flowering vegetative stage to the flowering stage. Attack period duration was therefore four and a half months per year. The peak of attacks was recorded in November with an attack rate of 88.02% in 2015 and 75.49% in 2016. No attack was recorded from February to mid-September, corresponding to the flowering, fruiting and post-harvest vegetative growth stages. This description of the attack process and the determination of *D. trifasciata* attack periods provides essential data for the implementation of an effective and sustainable control method of this species.

## 1. Introduction 

The cashew tree, *Anacardium occidentale* L., 1753 (Sapindales: Anacardiaceae), is a perennial plant cultivated and introduced in Côte d’Ivoire, circa 1960, in order to combat the threats of desertification and soil erosion in the north. From this ecological role, cashew tree cultivation switched to a socio-economic objective from the 1990s due to the growing demand for cashew nuts on the international market [1]. Raw cashew nut production has therefore developed dramatically. It rose from 19,000 tons in 1990 to more than 700,000 tons in 2015, that is, 25% of world production. Since 2015, Côte d’Ivoire has occupied the position of world leader in raw cashew nut production and export [2]. The value of raw cashew nut exports in 2015 in Côte d’Ivoire was estimated at more than $650 million. Cashew nut has become the third most important export product after cocoa and refined petroleum products in Côte d’Ivoire. Cashew nut is cultivated in northern, northwestern, northeastern, central and eastern Côte d’Ivoire. The cashew sector employs nearly 350,000 producers and feeds around 1.5 million people [3,4]. However, many challenges remain as this high cashew production is mainly due to an increase in the number of cashew producers and an expansion of orchards [5]. Nut yields from Ivorian orchards remain low. They range from 350 to 500 kg/ha [6] against a standard average yield ranging from 800 to 2000 kg/ha [7,8,9]. This low production is partly explained by the inexistence and/or non-compliance with good agricultural practices. This favors a proliferation of diseases and many insect pests such as the branch girdler *Diastocera trifasciata* (Cerambycidae: Lamiinae: Ceroplesini) (Fabricius, 1775) [10,11,12]. This pest, formerly called *Analeptes trifasciata*, is distributed in several West African countries. Its life cycle is one generation per year in 167–240 days. Eggs are laid singly in the severed branches of cashew trees. After hatching, first, instar larvae start feeding under the bark. Gradually, they penetrate the sapwood, making longitudinal galleries in which they live [13]. Mature larvae construct individual pupal chambers (55–135 mm long). Larval development occurs entirely within the cashew branches. Adults emerge through a circular exit hole (11–22 mm diameter) bored in the bark during the rainy season (from May to June). They then feed on the twigs and mate 123.33 ± 8.45 days after emergence [13]. 

In Côte d’Ivoire, damage caused by *D. trifasciata* was first reported by Brunck and Fabre [14] in Bouaké in a cashew tree orchard. However, since the nuts were not really saleable, no studies to reduce its damage have been carried out because they were useless [1]. Hence, the expansion of damage caused by the species in Côte d’Ivoire reached 55% cashew nut yield loss [15,16,17]. As a result, the population and the attacks have varied over the years [15,16].

According to previous studies carried out in Côte d’Ivoire, the emergence of adults of *D. trifasciata* and the fluctuation of adult population are linked to rainfall, relative humidity and average temperature [18]. In Nigeria, this periodic pattern of adults and their attacks has been exposed by Asogwa et al. [19]. No previous study has explained this variation in the attack rate over the year in Côte d’Ivoire. How does the attack occur? What could the factors triggering these attacks be? This study aims at knowing the attack behavior and the link between these and cashew tree phenological stages on *D. trifasciata* attacks in cashew tree orchards. 

## 2. Materials and Methods 

### 2.1. Study Area and Selection of Study Sites

The studies were conducted in the locality of Brobo, which is on the Bouaké–M’Bahiakro road, in central Côte d’Ivoire (004°49′29″ N and 07°38′18″ W) from October 2015 to September 2017. The climate is characterized by four seasons. There is a long, dry season from November to February marked by the harmattan (which blows continuously from December to February), a long rainy season from March to June, a short dry season from July to August and a short rainy season from September to October [20]. Rainfall ranges from 1200 to 1500 mm. The average temperature ranges from 20 to 27.5 °C. Relative humidity ranges from 57% to 85% [21]. Orchard selection criteria were the following: (i) having undergone *D. trifasciata* attack, (ii) having a surface area of at least 1 ha, (iii) being accessible all year round and (iv) being exempted from any insect management method for the entire duration of the study. Thus, three orchards with a surface area of 3 ha, at least 5 km away, were selected.

### 2.2. Experimental Layout

**Figure 1:**
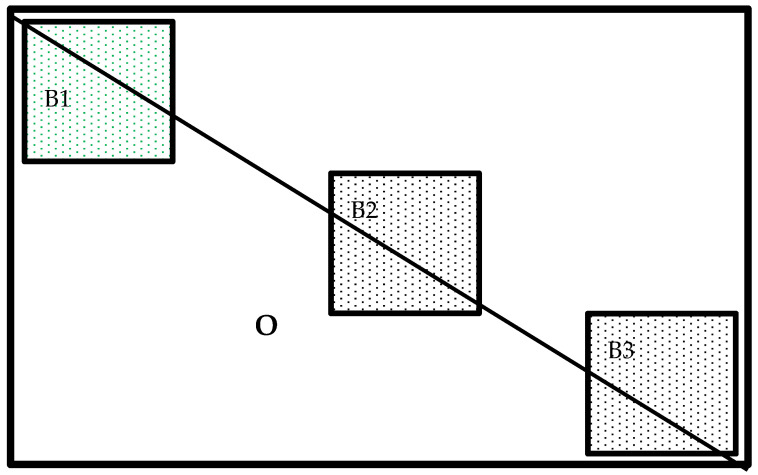
Experimental layout. O = orchard; B1 = Block 1; B2 = Block 2; B3 = Block 3.

In order to assess *D. trifasciata* damage, the diagonal method used by Ouédraogo [22] was applied. On one of the orchard diagonals, three non-contiguous blocks were determined, one (1) of which was in the middle and one (01) at each of its two ends (Figure 1). In each block, 51 cashew trees were randomly marked: that is, a total of 153 plants sampled per orchard each 15 days.

### 2.3. Attack Process and Assessment of Damage Caused by Diastocera trifasciata

**Figure 2:**
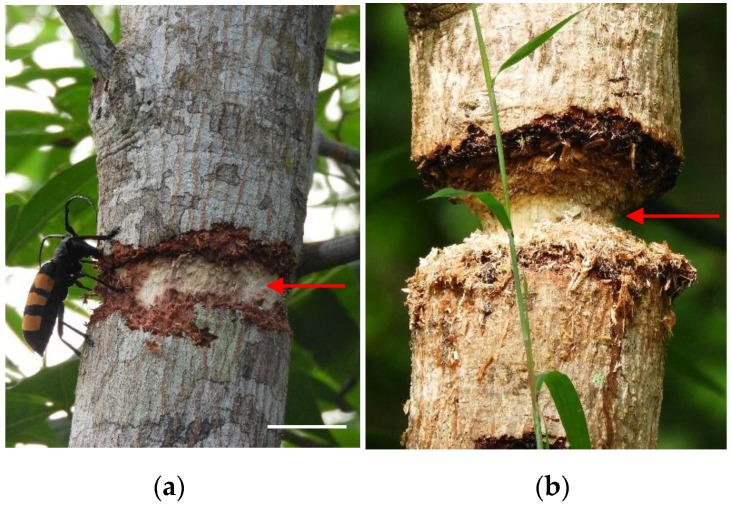
Levels of attack on branches used to estimate *D. trifasciata* damage intensity; (**a**) type 1 girdled branch: type 1 attacks were made up of girdled branches and/or stems where only the bark had been attacked, scale bar 3.6 cm; (**b**) type 2 deep girdling or cut branch: type 2 attacks included cut branches and/or stems whose attack had reached the sapwood, scale bar 2.83 cm. Red arrows indicate attacked area.

In order to describe the attack process and to estimate the time taken for the beetles to cut a branch, thirty-nine branches on all three orchards (13 branches per orchard) were monitored twice per day (7 am and 5 pm), every day from the beginning to the end of the attack. Each of the 153 sample trees were surveyed every 15 days to identify damage caused by *D. trifasciata*, and the number of attacked branches was recorded. The average infestation for each month was then determined. To assess the intensity, two types of attack were defined. Type 1 attacks were made up of girdled branches and/or stems where only the bark had been attacked. Type 2 attacks included cut branches and/or stems whose attack had reached the sapwood (Figure 2).

These attacked branches were counted per tree. Hanging branches on trees were removed with sickles and marked so that they were no longer recounted. The girth and length of the cut portion of each branch or trunk were measured with a decameter. We defined attack rate as the percentage of cashew trees attached by the girdler, which were calculated using the formula

(1) $$Ta=\frac{NAP}{TNSP}\times 100$$

*Ta* is the attack rate, *NAP* is the number of attacked plants and *TNSP* is the total number of sampled plants.

We defined intensity of attack as the number of girdled and cut branches per cashew tree sampled using the following formula:

(2) $$Ia=\frac{NCB}{TNSP}$$

*Ia* is intensity of attacks, *NCB* is the number of cut branches and *TNSP* is the total number of sampled plants. 

### 2.4. Effect of Biotic Factors on D. trifasciata Attacks 

These factors are the phenological stages of cashew tree. An annual cashew tree production cycle is marked by the following phenological stages: the pre-flowering vegetative growth stage (Pfv), which takes place from September to November; the flowering (Flow), which takes place in December and January; the fruiting phase (Fru) from February to April; and the post-harvest vegetative growth stage (Phv) from May to August. The evolution of these stages was noted each month.

### 2.5. Statistical Analysis 

All data were processed using Statistica 7.1 software, StatSoft version [23]. Attack rates were subjected to the Kruskal–Wallis non-parametric test to compare attack levels from one phenological stage of the cashew tree to another.

## 3. Results

### 3.1. Process of Branch and Stem Attack by Diastocera trifasciata 

**Figure 3:**
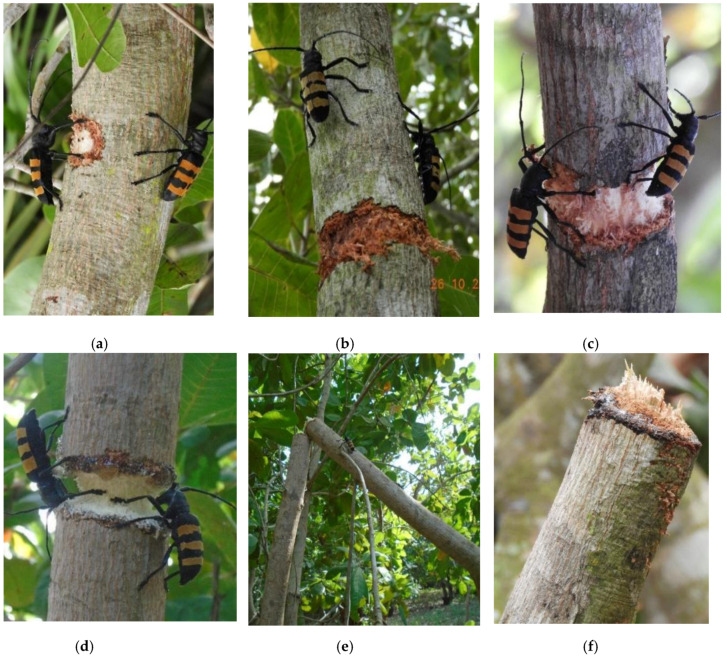
Process of cashew tree branches attack by a couple of *Diastocera trifasciata*. (**a**) Start of attack at bark level, scale bar 20.4 mm; (**b**) deep attack of the bark, scale bar 18.5 mm; (**c**) attack on sapwood, scale bar 13.8 mm; (**d**) very deep attack, scale bar 16 mm; (**e**) end of attack and breakage of the branch, scale bar 16 mm; (**f**) side view of an attack area on a cut branch, scale bar 17 mm.

The attack was carried out by a mating pair of adults. The male and female moved to either side of the selected branch or stem and started gnawing the branch/stem in a circular way. The attack was carried out gradually from the outside to the inside, starting with the bark and the cambium (Figure 3a–c). During the process, both individuals occasionally stopped the attack either to mate or to rest (Figure 3b). Sometimes it was rather the male who rested while the female continued the girdling, and vice versa. When the girdling reached the sapwood, a yellowing of the leaves borne by the attacked branch was noted. The attack area then took the form of a “V” inclined on the left or right side depending on whether we looked at the left or right side of the attack area, respectively (Figure 3d).

When the branch was completely cut (Figure 3e), the female laid her eggs on it. Then, the pair attacked a new branch, and the attack process described above began again. The cut branches lay strewn on the ground of the orchards or remained attached to the trees. Average time for a pair of beetles to sever a branch was 12.36 ± 3.05 days (mean ± SD). The girth and length of attacked branches varied from 8.5 to 35 cm (18.04 ± 2.43 cm) and from 1 to 4.9 m (3.08 ± 0.75), respectively. Some branches that had resisted attacks in orchards were recorded. The base of each cut branch showed a smooth, regular cut as if it were cut with a saw (Figure 3f). 

### 3.2. Assessment of Attacks of Adults of D. trifasciata on Cashew Trees

**Figure 4:**
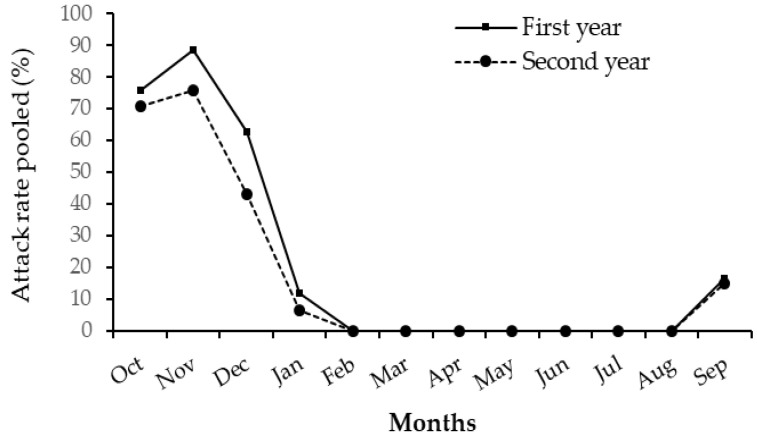
Monthly evolution of attacks across all sites per year. First year: October 2015–September 2016; Second year: October 2016–September 2017.

The attacks started in mid-September with an average attack rate of 16.33% across all sites. These attacks progressively evolved, reaching the peak of 88.02% (attack rates) in November of the first year and 75.49% (attack rate) in the second one. Attacks started falling until January with a rate of 11.83% before reaching zero from February to August. A single attack period of four and a half months from mid-September to January each year can be distinguished in *D. trifasciata*. The lowest attack rates were recorded in September and January and the highest ones in October and November (Figure 4).

### 3.3. Assessment of the Intensity of Attacks by Adults of D. trifasciata on Cashew Trees 

Over the two years of study, the number of cut branches was 6044, distributed as follows: 2959 for site 1, 1679 for site 2 and 1406 for site 3. On average, during the ten months of attacks, there were 0.97 ± 1.44 branches per tree that were cut at site 1 (between 0.06 and 2.97). At site 2, there were 0.55 ± 0.99 per tree (between 0.05 and 1.25). At site 3, 0.46 ± 0.88 branches were cut per tree (between 0.04 and 0.88).

### 3.4. Relationship between Attack of D. trifasciata and Cashew Tree Phenological Stages

**Figure 5:**
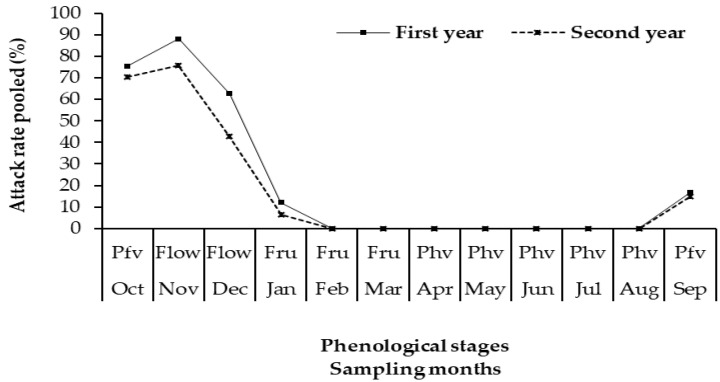
Relationship between attack of *D. trifasciata* and cashew tree phenological stages per year; First year: October 2015–September 2016; Second year: October 2016–September 2017. Pre-flowering vegetative growth stage = Pfv; Flowering stage = Flow; Fruiting = Fruc; Post-harvest vegetative growth stage (Phv).

Attacks by *D. trifasciata* varied with host phenological stages (Figure 5). Indeed, the attacks started when the trees were in the pre-flowering vegetative growth stage. When the trees started flowering, the attacks decreased sharply until they stopped at the fruiting and post-harvest vegetative growth stages (Figure 5). Attacks were highest at the pre-flowering vegetative growth stage. The Kruskal–Wallis test indicated that the variations in the attack rate during the phenological stages of cashew trees were statistically significant (H (3, N = 11,015) = 5369.484; *p* < 0.01).

## 4. Discussion

The process of *Diastocera trifasciata* attack on branches described in this study is similar to that observed in the genus *Oncideres* Lepeletier de Saint Fargeau & Audinet-Serville (Cerambycidae: Lamiinae) [24,25,26,27,28]. However, differences were observed in the number of individuals attacking the branch and the size of the branches attacked. In *D. trifasciata*, the attacks were carried out by a mating pair, while in all *Oncideres* species, girdlers in America, they are carried out only by an individual female [24,28,29,30]. Unlike some species of the genus *Oncideres* such as *O. impluviata* (Germar, 1842), *O. occularis* Thomson and *O. humeralis* Thorms, adults of *D. trifasciata* cut large branches (18.04 ± 2.43 cm during 12.39 ± 3.05 days). The selection of large branches might be linked to the size of adults. Indeed, the diameter of the branches girdled by *Oncideres spp*. ranges from 1.30 to 6.28 cm compared to 18.04 ± 2.43 cm in *D. trifasciata*. In addition, adult *Oncideres spp*. measure 1.15 to 2.80 cm compared to adults of *D. trifasciata* (35–50 cm). Many species of the tribe Onciderini (Cerambycidae) show great specificity toward the host by selecting branches with the desired characteristics [29,30,31].

The high attack rates recorded (70 to 88%) show the danger that this species represents for cashew tree cultivation in the places where it prevails. In Côte d’Ivoire, previous work had noted the highly harmful nature of *D. trifasciata* [15,16,32]. Without intervention, the number of eggs laid increases with the number of cut branches, and this results in an increase in the population of the future generation. Unfortunately, post-attack branch management is not correctly practiced in Côte d’Ivoire, and in the sub-region, due to the lack of knowledge of the biology and phenology of the species [15,19]. As a result, cashew producers only notice the beetles in the adult stage during the attack period. However, the larvae and pupae of the insect develop in cut branches. This set of constraints amplifies the attacks year over year and accentuates the establishment of the population in a sustainable manner in orchards in Côte d’Ivoire.

The first attacks of the species were recorded in mid-September when the pre-flowering vegetative growth of trees started. They increased until they reached the peak in November, with trees at the same stage. Conversely, at the flowering stage, the attacks fell until they reached zero at the fruiting stage from January. The triggering factor for the attacks might be the physiological state of cashew trees with favorable abiotic conditions. During this period, the sap might be richer in nutrients favorable to the development of larvae. This observation was also made by Uribe-Mú and Quesada [24]. These authors indicated that adult females of *Oncideres albomarginata chamela* Chemsak & Giesbert (Coleoptera, Cerambycidae) preferentially cut *Spondias purpurea* L. (Anacardiaceae) branches before the reproductive period of the tree. They showed that the trees in that period had reached the maximum concentration of non-structural carbohydrates and nitrogen, significant in larvae development. In contrast, certain species of the genus *Oncideres* attack and cut the branches of healthy host plants, when the peak of flowering and leaf bud break is reached [30,33,34,35]. The species might therefore synchronize its cycle with that of its host plant for its reproduction. In contrast, the gradual decline in attacks from flowering to their complete absence from fruiting might be due to climatic conditions becoming more and more hostile to adult insects [18]. During this (dry) period, the adults die and are replaced by larvae that develop in previously cut branches. They entirely develop in the branches until adult emergence in the rainy season in May [13,18,36]. During this dry period, a possible control method against larvae might be as follows: systematic removal and burning of all branches in which individuals of the next generation develop in larval form. This physical control is more accessible, not expensive for the cashew growers and spares cashew nuts from any pesticide residues.

## 5. Conclusions

This study has shed light on the process of attack on branches and stems by the pest *Diastocera trifasciata*. These attacks are always carried out by a male and a female pair. On average, a beetle pair required 12.36 ± 3.05 days to cut a 18.04 ± 2.43 cm girth branch. A four and a half month attack period was observed from mid-September to January. Between February and August, no attacks were recorded on cashew trees. Maximum attacks were recorded in October and November. It also appears that the factors favorable to *D. trifasciata* attacks are the pre-flowering vegetative growth stage and the rainy season. Adult emergence and attack coincides with host phenology, and the latter is associated with climate. 

Further studies should be done in order to know the relationship between the attacks and other biotic variables such as tree height, branch length, tree vigor or bark thickness and climatic factors which are known to have an impact on cerambycids’ reproductive strategies. In addition, studies on the susceptibility of cashew trees to attack would be necessary to determine resistant varieties. This could be a good information base for producers in understanding and managing beetle attacks. 

Knowledge of attack periods and peaks is a necessary basis for developing any management method for the species by providing information on ideal intervention periods. Our study indicates that growers have a large window (February to May) during which to remove girdled branches from orchards and destroy developing larvae and pupa, in order to reduce adult densities and future damage by *D. trifasciata.*
